# The Effect of Oat Hay, Alfalfa Hay, and Their Combined Diets on the Morphology and Function of the Pancreas in Preweaning Yak Calves

**DOI:** 10.3390/ani13020293

**Published:** 2023-01-14

**Authors:** Yang Jiao, Yanan Zhou, Shujie Liu, Deyu Yang, Jilan Li, Lu Sun, Zhanhong Cui

**Affiliations:** 1 Qinghai Academy of Animal Husbandry and Veterinary Sciences, Qinghai University, Xining 810016, China; 2 Ministry of Agriculture and Rural Affairs Key Laboratory of Animal Nutrition and Forage-Feed of Grazing Yak and Tibetan Sheep in Qinghai-Tibetan Plateau, Xining 810016, China; 3 Yak Engineering Technology Research Center of Qinghai Province, Xining 810016, China; 4 Key Laboratory of Plateau Grazing Animal Nutrition and Feed Science of Qinghai Province, Xining 810016, China; 5 College of Agriculture and Animal Husbandry, Qinghai University, Xining 810016, China

**Keywords:** preweaning yak calves, oat hay, alfalfa hay, pancreas development, metabolomics

## Abstract

**Simple Summary:**

The purpose of this study was to investigate the effects of different source forages fed to preweaning yak calves on growth performance, pancreatic morphology, and functional development. In the experiment, we performed three different sets of experimental treatments by feeding tests, collected pancreatic tissues and recorded their organ indices at the end of the experiment, collected pancreatic tissue samples to observe morphological structures by tissue sectioning, determined the activity of main digestive enzymes and hormone levels in the pancreas by enzyme-linked immunosorbent assay, and obtained metabolic pathways and their small-molecule differential metabolites by nontargeted metabolomics techniques. The endocrine and exocrine divisions of the pancreas, which functions as a metabolic organ, are involved in the control of glucose and the organism’s digestive metabolism. We discovered that feeding preweaning yak calves a combination of oat hay and alfalfa hay was better for the morphological and functional development of the pancreas than feeding either hay alone. This is essential for the early development of the pancreas in yak calves and serves as a critical resource for their healthy growth and scientific feeding.

**Abstract:**

In this study, we used a combination of animal nutrition and nontargeted metabolomics to investigate the effects of feeding different sources forages rations on the morphology and function of the pancreas in preweaning yak calves, providing theoretical guidance and important references for the healthy and high-quality rearing of yak calves. At 45 days old, 21 yak calf males were divided into OP, AP, and AOP groups, with seven animals in each group, which were fed with oat hay, alfalfa hay, and mixed oat and alfalfa hay, respectively. Five calves from each group were selected randomly to slaughter after a pretest period of 21 days and the official period of 120 days, when the average daily feed intake reached 1 kg. During the test, the growth and pancreas weight of yak calves were recorded, and the morphology and function of the pancreas tissues were determined using tissue sectioning methods, enzyme-linked immunosorbent assay (ELISA) tests, and nontargeted metabolomics strategies. The results showed that the body weight and pancreatic organ index of yak calves in the AOP group were significantly higher than those of the AP and OP groups. Compared to the AP and OP groups, the AOP group had considerably lower ratios of the area of the pancreatic endocrine component and overall percentage of that section of the organ, and the AOP group increased pancreatic amylase activity and a higher concentration of growth inhibitor. The AP group had significantly higher levels of the differential metabolites L-ascorbic acid, spermidine, spermine, and dopaquinone in the glutathione, β-alanine, and tyrosine metabolic pathways than the OP group. The AOP group had significantly lower levels of the differential metabolites spermine and phenylacetylglycine in the glutathione and phenylalanine metabolic pathways than the AP group. In summary, compared to feeding oat or alfalfa hay alone, combined feeding oat hay and alfalfa hay is more beneficial to promote the morphological and functional development of the pancreas in preweaning yak calves, so as to enhance the digestion and absorption of nutrients in the diet and maintain the positive regulation of blood glucose levels. This provides an important basis for the optimized forage supply of healthy and high-quality rearing in preweaning yak calves.

## 1. Introduction

Yaks have great ecological and economic value as the primary animal product farmed by herders on the Qinghai plateau [1]. The core of the premium yak business is the calf, and the calves’ early breeding is essential to their future growth and development. [2]. The traditional way of feeding with milk may cause problems such as low immunity and slow growth in calves [3]. Guan Jiuqiang et al. found that yak calves were given milk replacer powder to replace their mother’s milk when they were 3 months old or younger [4]. Compared to conventional grazing calves, their body weight was much higher, dramatically promoting yak calves’ growth and development, as well as increasing the mother yaks’ reproductive productivity. When nursing was replaced with milk replacer and supplementary feeds for suckling in 2-month-old yak calves, Chai Shiming et al. observed that the body weight of yak calves rose dramatically between 60 and 180 days of age [5], which improved the productive performance of yaks and the economic efficiency of local herders. Feeding diets with high or low concentrate-to-forage ratios will have different effects on yak growth and development. Li Yan et al. found that the average daily weight gain [6], apparent feed digestibility, and rumen microbial protein content of yak bulls at the late fattening stage were significantly higher than other groups when the ratio of dietary concentrate to forage was 65:35. According to research by Contreras et al. [7], feeding post-weaned calves a combination of alfalfa and oat hay can enhance dietary nitrogen efficiency and growth performance through influencing rumen fermentation, which provides advantages for growth and increased feed digestion. Feeding different sources of forage diets on top of supplemental concentrates can have different effects on the growth and development of yaks. When adult yaks were around 3 years old, Yang et al. discovered that supplementing their diet with oat hay improved their daily weight gain [8], as well as reduced their weight loss and energy expenditure in the cool season, in addition to raising the herders’ financial rewards. Cui et al. found that feeding milk replacer and supplemental alfalfa hay to pre-weaned yak calves could alter the gastrointestinal microbial community [9], thus contributing to improved digestion and absorption, growth, and immune function in yak calves. Hao Wenjun et al. found that, on the basis of feeding concentrate and corn silage [10], there were significant differences in rumen bacterial compartments in the alfalfa hay and oat hay groups compared to the wheat straw group. According to Xia Hongze et al. [11], the fermentation gas production, dry matter degradation rate, and volatile fatty acid content of yak rumen were significantly higher than those of other combinations, and the proportion of roughage fed to them was more reasonable, when the grading index of the combination of alfalfa hay and oat hay reached 6.40. It is evident that feeding various oat hay and alfalfa hay to yak calves together with additional milk replacer and concentrates has varying impacts on their growth and development.

As an important secretory organ of the body, the pancreas is composed of two main parts: the endocrine and exocrine parts [12]. Moreover, the pancreas secretes mainly pancreatic amylase, trypsin, chymotrypsin, pancreatic lipase, insulin, glucagon, growth-inhibitory hormone, and pancreatic polypeptides [13]. The digestive and absorption processes are aided by the enzymes released in the alkaline pancreatic juice. The pancreas is an important organ for controlling digestion and food metabolism because it secretes islet hormones into the glandular follicles to regulate protein and fat metabolism and keep glucose levels in a healthy range [14]. Yang et al. found that feeding yaks a high energy nutrition level of 6.90 MJ/kg resulted in significantly higher plasma concentrations of insulin (INS) and insulin-like growth factor 1 (IGF-1) [15], which more effectively controlled and enhanced yaks’ growth performance. By providing pre-weaned yak calves with concentrates and alfalfa hay, Wu et al. were able to increase pancreatic α-amylase activity [16]. This in turn affected the calves’ concentration of ruminal propionate, which in turn caused an upregulation of ruminal cholecystokinin in the pancreatic secretory pathway. Schneeman et al. discovered that additional feeding of oat and alfalfa to young rats boosted amylase activity and insulin content in the pancreas compared to the control group [17], boosting their digestion and metabolism, as well as having some antiaging benefits. This demonstrates how varied feedings of oat and alfalfa hay may promote the release of pancreatic enzymes and hormones, as well as have an impact on pancreas growth and development. Fewer studies have been conducted on the effect of feeding oat hay, alfalfa hay, and their mixture on the pancreas of pre-weaned yak calves on top of concentrate. The present study was conducted to investigate the effects of feeding different forage sources on the growth performance and pancreatic development of yak calves by feeding oat hay, alfalfa hay, and mixed oat and alfalfa hay in addition to concentrate, to offer a theoretical foundation for high-quality growth and development and scientific feeding management of yak calves throughout their development.

## 2. Materials and Methods

### 2.1. Test Animals

This study involved 21 yak bull calves with an average weight of 36.5 ± 1.0 kg in good health, at about 45 days of age, with seven animals each randomly split into OP, AP, and AOP groups. In addition to milk replacer and concentrate, the three groups were given oat hay, alfalfa hay, and blended oat and alfalfa hay. The animal tests were conducted in the pastures of the Plateau Ecological Animal Husbandry Science and Technology Demonstration Park of Haibei Tibetan Autonomous Prefecture in Qinghai Province, and the sample measurements were performed in Key Laboratory of Plateau Grazing Animal Nutrition and Feed Science of Qinghai Province, Qinghai Academy of Animal Husbandry and Veterinary Sciences in Qinghai University.

### 2.2. Feeding and Management

The composition and nutritional levels of milk replacer, concentrate, oat hay, and alfalfa hay in the rations in the feeding trials are shown in Table 1. Following randomization, milk replacer and concentrate were given to all three groups. The OP group was given oat hay, the AP group was given alfalfa hay, and the AOP group was given a 1:1 mixture of oat and alfalfa hay. Each group received the same quantity of food with a concentrate-to-roughage ratio of 3:7. Following the 21-day pretesting phase, the 120-day formal test period began. The milk replacer was mixed with boiling water at 100 °C in the ratio of 1:5 and cooled to 42 °C. The milk replacer was fed in three periods: morning, afternoon, and evening. Milk replacer, concentrate, and forage were fed at 0.48 kg/day, 0.06 kg/day, and 0.14 kg/day on the first day, and then increased by 15 g, 30 g, and 70 g per week. Throughout the experiment, yak calves were maintained in individual pens, given unrestricted access to food and water, and regularly cleaned and disinfected.

### 2.3. Sample Collection

After the feeding test and a 12 h fast, five calves from each group were randomly chosen for the slaughter test, and the abdominal cavity of the yak calves was opened for dissection. When collecting pancreatic tissue samples, the samples were rinsed with saline, and trimmed to 1.5 cm, 1.5 cm, and 2 cm in length, width, and height, respectively. The samples were put into 50 mL centrifuge tubes and 5 mL freezer tubes, which were each filled with samples at around two-thirds of the capacity. The centrifuge tubes also included 4% paraformaldehyde to preserve the tissues. The samples were collected and placed immediately into a liquid nitrogen tank set at −196 °C before being moved to an ultralow-temperature refrigerator at −80 °C.

### 2.4. Index Determination and Method

#### 2.4.1. Growth and Development of the Pancreas

Yak calves were weighed, and the data were recorded prior to the slaughter test; the organ index was calculated after removing the pancreatic tissue for weighing. Pancreatic index (%) = weight of pancreas/pre-slaughter weight × 100.

#### 2.4.2. Morphological Development of the Pancreas

An inverted imaging microscope was used to study the pancreatic tissue slices, and four randomly chosen fields of view in each section were photographed. Using Image-Pro Plus 6.0 software (Media Cybernetics, Rockville, MD, USA) and a uniform field of view of 1 × 10^5^ μm^2^, the area of the pancreas in its field of view was measured, calculated, and recorded. The total area ratio of the exocrine, total area ratio of the endocrine, and area ratio of the endocrine part of the pancreas were also calculated. Overall pancreatic endocrine part area share (%) = area of the pancreas endocrine portion divided by the organ’s total area multiplied by 100. Total pancreatic exocrine portion area share (%) = total pancreatic area multiplied by the exocrine component of the pancreas. The area of the endocrine portion of the pancreas can be divided by the area of the exocrine portion of the pancreas to obtain the area share of the pancreatic endocrine to exocrine part (%).

#### 2.4.3. Functional Growth of the Pancreas

Pancreatic specimens were weighed at around 0.2 g on an electronic scale. PBS buffer was added to the sample to dilute it 10-fold, and the sample was thoroughly mixed with a vortex shaker. The supernatant was meticulously collected and distributed after centrifugation at 2500 r/min on a frozen high-speed centrifuge for 20 min. To the enzyme-linked immunosorbent assay (ELISA) kit plate, 50 µL of the standard sample, 10 µL of pancreatic supernatant sample, 40 µL of diluent (the sample diluted five times in total), and 100 µL of the enzyme standard reagent were added. After 60 min at 37 °C in the oven, five washes later, 50 µL of each color developer A and B are added, and the color was developed for 15 min while being shielded from light. Next, 50 µL of the termination solution was added, and an enzyme calibrator was used to measure the absorbance OD value at 450 nm. The primary digestive enzyme activity and hormone contents in the pancreas were determined after the assay findings were displayed as a standard curve to develop a linear regression equation for the samples. All ELISA kits were purchased from Jiangsu Enzymatic Biotechnology Co., Ltd. (Yancheng, China). The experiment’s enzyme activity and hormone concentrations were all determined using the same approach as before. The intra-group and inter-group coefficients of variance were under 10% and 15%, respectively, and the sensitivity was less than 1.0 U/L.

#### 2.4.4. Metabolomics of the Pancreas

A 100 mg sample of pancreatic tissue was removed, crushed using liquid nitrogen, and put in an Eppendorf (EP) tube. After adding 500 µL of an 80% methanol solution, the sample was mixed well with a vortex mixer, placed in a water bath for 5 min, and then spun at a high speed for 20 min at 4 °C. A certain amount of the supernatant after centrifugation was diluted with mass spectrometry water until the methanol level reached 53%. A certain amount of supernatant was collected and transferred into the liquid chromatograph/mass spectrometer (LC–MS) (Thermo Fisher Scientific, Waltham, MA, USA) for analysis after being frozen and centrifuged again for 20 min. The experimental procedures for the aforementioned metabolomics technologies were acquired from Novogene Co., Ltd. (Beijing, China).

### 2.5. Data Processing and Statistic Analysis

The data on the growth, morphology, and functional development of the pancreas were initially analyzed using Excel 2010 (Microsoft, Redmond, WA, USA), and a statistical *t*-test was performed using SPSS 25.0 software (SPSS Inc., Chicago, IL, USA); the data were expressed as the “mean ± standard deviation”, with *p* ≤ 0.05 being the threshold for the significance of differences. Meta X 21.0 software from BGI Genomics Co., Ltd. (Shenzhen, China) was used to analyze the pancreas metabolomics data in order to determine the variable projective importance (VIP value) of metabolites, and SPSS 25.0 software was used to analyze the data and the fold change (FC) value for each metabolite content. After establishing the threshold values, the Kyoto Encyclopedia of Genes and Genomes (KEGG) database was used to search for small-molecule differential metabolites, which were then examined and annotated.

## 3. Results

### 3.1. Effects of Different Forage Source Diets on the Growth and Development of the Pancreas in Preweaning Yak Calves

As shown in Table 2, the final body weight and pancreatic organ index in the AOP group were significantly greater than in the AP group (*p* < 0.05), while the final body weight, pancreatic weight, and pancreatic organ index in the AOP group were significantly higher than in the OP group (*p* < 0.05).

### 3.2. Effects of Different Forage Source Diets on the Morphological Development of the Pancreas in Preweaning Yak Calves

**Figure 1:**
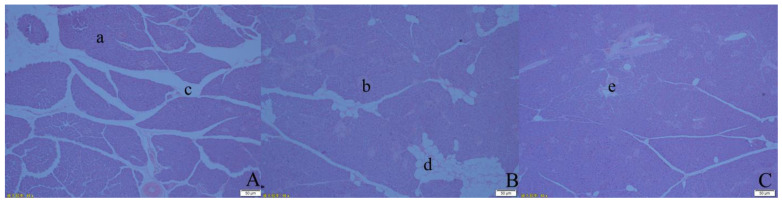
The pancreas morphological development in three different groups of yak calves: (**A**) the OP group of yak calves at 10×; (**B**) the AP group of yak calves at 10×; (**C**) the AOP group of yak calves at 10×. As seen in the images, the letters “a”, “b”, “c”, “d”, and “e” stand for glandular vesicles, pancreatic islets, interstitial spaces, fat vacuoles, and capillaries, respectively.

As shown in Figure 1, the density of the cell arrangement of pancreatic tissue increased sequentially from OP to AP to AOP groups, and the area of glandular vesicles and islets increased, while the number of inter-tissue spaces, fat vacuoles, and capillaries decreased.

As shown in Table 3, the AOP group’s total area ratio of the pancreas exocrine portion was substantially greater than that of the OP group’s (*p* < 0.05). The AP group’s total area ratio of the pancreatic endocrine portion and the area ratio of the pancreatic endocrine portion were both substantially larger than those of the AOP group (*p* < 0.05). Additionally, the OP group considerably outperformed the AOP group in terms of both the area ratio of the pancreatic endocrine portion and the overall percentage of the endocrine portion’s area (*p* < 0.05).

### 3.3. Effects of Different Forage Source Diets on the Development of Pancreatic Function in Preweaning Yak Calves

As shown in Table 4, chymotrypsin, pancreatic lipase, and pancreatic amylase activities were all substantially greater in the AOP group than the OP group (*p* < 0.05). The AOP group’s pancreatic amylase activities were substantially greater than those of the AP group’s (*p* < 0.05). The AP group had considerably greater pancreatic amylase activity than the OP group did (*p* < 0.05).

As shown in Table 5, glucagon, insulin, pancreatic polypeptide, and growth inhibitor concentrations were all considerably greater in the AOP group than in the OP group (*p* < 0.05). The AOP group’s concentration of growth inhibitor was substantially greater than that of the AP group’s (*p* < 0.05). Glucagon, pancreatic polypeptide, and growth inhibitor concentrations in the AP group were much greater than those in the OP group (*p* < 0.05).

### 3.4. Effects of Different Forage Source Diets on the Metabolomics of the Pancreas of Preweaning Yak Calves

**Figure 2:**
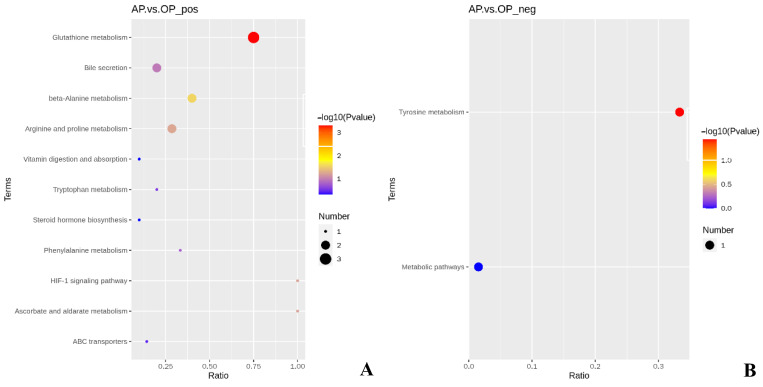
(**A**) KEGG metabolic pathway of AP vs. OP in positive ion mode; (**B**) KEGG metabolic pathway of AP vs. OP in negative ion mode. AP represents the group fed alfalfa (n = 5). OP represents the group fed oat (n = 5).

As shown in Figure 2, there were 13 KEGG metabolic pathways enriched by differential metabolites in the AP group compared to the OP group.

As shown in Table 6, the content of the differential metabolites L-ascorbic acid, spermidine, and spermine in the glutathione metabolic pathway was significantly higher in the AP group compared with the OP group (*p* < 0.05). In the beta-alanine metabolism pathway, the levels of the differential metabolites spermidine and spermine were significantly increased (*p* < 0.05). In the tyrosine metabolic pathway, the levels of the differential metabolite dopaquinone were significantly increased (*p* < 0.05).

**Figure 3:**
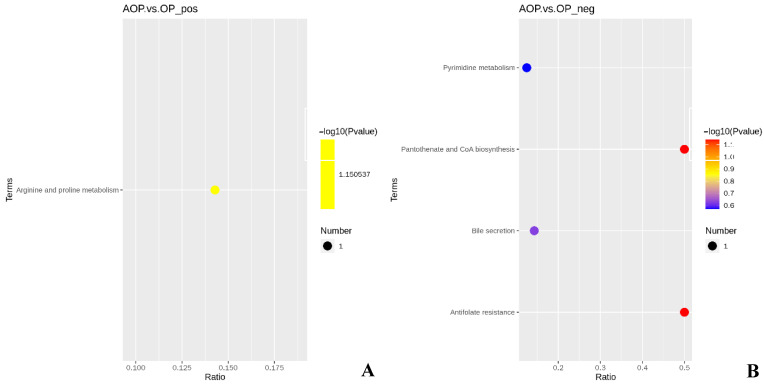
(**A**) KEGG metabolic pathway of AOP vs. OP in positive ion mode; (**B**) KEGG metabolic pathway of AOP vs. OP in negative ion mode. AOP represents the group fed a mixture of oat and alfalfa (n = 5). OP represents the group fed oat (n = 5).

As shown in Figure 3, there were five KEGG metabolic pathways enriched by differential metabolites in the AOP group compared to the OP group.

As shown in Table 7, the AOP group had nonsignificant levels of relevant differential metabolites in all their metabolic pathways compared to the OP group (*p* > 0.05).

**Figure 4:**
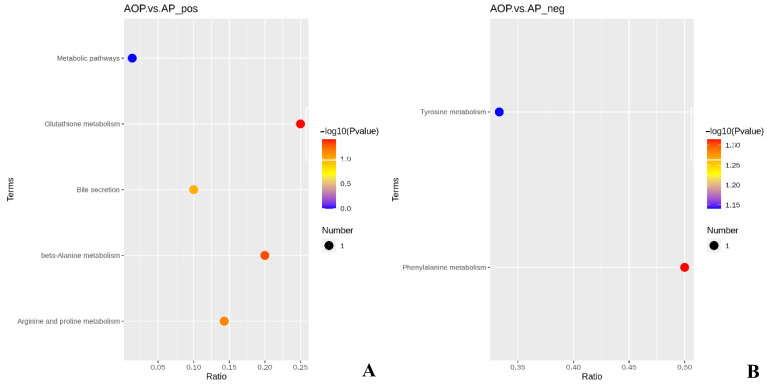
(**A**) KEGG metabolic pathway of AOP vs. AP in positive ion mode; (**B**) KEGG metabolic pathway of AOP vs. AP in negative ion mode. AOP represents the group fed a mixture of oat and alfalfa (n = 5). AP represents the group fed alfalfa (n = 5).

As shown in Figure 4, there were seven KEGG metabolic pathways enriched by differential metabolites in the AOP group compared to the AP group.

As shown in Table 8, compared with the AP group, the AOP group showed a significant decrease in the content of the differential metabolite spermine in the glutathione metabolic pathway (*p* < 0.05) and a significant decrease in the content of the differential metabolite phenylacetylglycine in the phenylalanine metabolic pathway (*p* < 0.05).

## 4. Discussion

### 4.1. Effects of Different Forage Source Diets on Growth Performance of Preweaning Yak Calves

The results showed that feeding a mixture of oat and alfalfa hay increased body weight, pancreas weight, and its organ index in preweaning yak calves compared to feeding a single oat hay or a single alfalfa hay. Zou et al. replaced some of the alfalfa hay with oat hay or corn silage and found that yak calves fed a mixture of oat and alfalfa hay 7 days after weaning had higher body weight and daily gain than animals fed other forage combinations [18]. Tang Qi et al. concluded that pancreatic development can promote pancreatogenesis and its progenitor cell differentiation [19], which facilitates the digestive role of pancreatic juice secreted by the glandular vesicles. Preweaning yak calves had greater benefits from receiving a combination of oat and alfalfa hay than they did when receiving only oat or alfalfa hay, and the yak calves showed superior growth advantages and pancreatic development as a result.

### 4.2. Effects of Different Forage Source Diets on the Morphological Development of the Pancreas in Preweaning Yak Calves

According to research by Twersky et al., non-purified fibers such as whole oat or whole alfalfa decreased pancreatic amylase and pancreatic protease activity and may be a factor in the pancreatic exocrine insufficiency [20]. When Svensson et al. compared the capillary areas in the endocrine and exocrine parenchyma of the pancreas, they discovered that the capillary volume in the islets [21], the endocrine portion of the pancreas, was roughly 3.5%, whereas the capillary volume in the exocrine portion of the pancreas was significantly lower at 2%. The absence of lymphatic capillaries and the comparatively narrow capillary lumen in pancreatic islets may be the cause of the discrepancy between these characteristics. As a result, the exocrine portion of the pancreas accounts for a significant total percentage of its area, while the endocrine portion accounts for a tiny total percentage. Of course, the ratio of the endocrine division to the exocrine division falls as the area of the endocrine division increases. Calvert et al. found that, in rats fed diets without added fiber, with added viscous fiber, and with added insoluble fiber (oat and alfalfa), the activity of major digestive enzymes such as pancreatic protease in the pancreas tended to be higher, promoting the growth and development of the exocrine part of the pancreas [22]. In this study, feeding a mixture of oat and alfalfa hay increased the total percentage of the exocrine part of the pancreas in preweaning yak calves compared to feeding a single oat hay or a single alfalfa hay. The proportion of the endocrine part of the pancreas and the proportion of the internal and external areas were significantly lower in the group fed a mixture of oat and alfalfa hay compared to the other groups. This shows that feeding oat and alfalfa hay had a greater effect on the exocrine part of the pancreas of preweaning yak calves and extremely strongly promoted the development of the pancreas and the secretion of its digestive enzymes, which had a positive effect on the enhancement of the digestive and metabolic capacities of preweaning yak calves.

### 4.3. Effects of Different Forage Source Diets on the Development of Pancreatic Function in Preweaning Yak Calves

According to research by Emanuela et al., eating whole oat or whole alfalfa decreased the activity of the pancreatic enzymes pancreatic amylase, pancreatic protease, alloxan, and pancreatic lipase in the dietary fiber of people [23]. When compared to nonpure fibers, Solka-purified Floc’s fibers dramatically reduced pancreatic lipase activity, lowered chymotrypsin dependency, and decreased plasma cholesterol and triglyceride levels. According to research by Zhao et al., feeding dairy cows a blend of oat and alfalfa hay high in digestible fiber and high-quality protein can enhance cow performance and feed digestibility [24]. Li Ru discovered that pancreatic release of digestive enzymes may enhance ruminant small intestine digestion and absorption of small-molecule nutrients, as well as support the passage of pancreatic enzymes through the duodenum to carry out typical digestive tasks [25]. In this study, it was shown that feeding a mixture of oat and alfalfa hay enhanced the activities of pancreatic amylase, pancreatic protease, pancreatic lipase, and pancreatic lipase in suckling yak calves compared to eating a single oat hay or a single alfalfa hay. This is in line with the finding that feeding a combination of oat and alfalfa hay was more advantageous in fostering the growth and development of the exocrine component of the pancreas. It demonstrates that providing mixed hay together with oat and alfalfa can more successfully encourage the digestion and absorption of nutrients in the diet by the primary digestive enzymes of the pancreas, which is better for the growth and development of young yak calves.

Habtamu et al. found that fiber in whole oat or whole alfalfa diets has negative effects as antinutritional factors or non-nutritional compounds that have negative health effects [26], i.e., antinutrients. Subandi et al. found that the response of antinutrients to starchy foods can reduce insulin levels and blood glucose levels [27], which may exacerbate pancreatic insufficiency and poor digestion in calves. Andersen et al. found that nutrients ingested by the body can regulate the secretion of pancreatic hormones [28], while the interaction of insulin, glucagon, growth-inhibitory hormone, pancreatic polypeptide, and extra-pancreatic hormones achieves the regulation of body metabolism by affecting blood glucose, appetite, gastric emptying, and intestinal motility. In this study, feeding a mixture of oat and alfalfa hay increased the levels of insulin, glucagon, growth-inhibitory hormone, and pancreatic polypeptide in preweaning yak calves compared to feeding a single oat or alfalfa hay, and feeding a mixture of both helped to maintain the balance of blood glucose metabolism in the body and provided a strong guarantee for the stability of the internal environment of preweaning yak calves.

### 4.4. Effects of Different Forage Source Diets on the Metabolomics of the Pancreas of Preweaning Yak Calves

In this study, there were significant differences in three metabolic pathways in the group fed alone alfalfa compared to the group fed alone oat. In the glutathione metabolic pathway, the levels of the differential metabolites L-ascorbic acid, spermidine, and spermine were elevated. According to Zhang’s research, L-ascorbic acid, which is essential for the body’s metabolic and antioxidant functions [29], cannot be directly produced or stored in animals and must instead be consumed through the consumption of plant-based nutrients. Dehydroascorbate reductase (DHAR) catalyzes the reduction of reduced glutathione during the L-ascorbic acid metabolic cycle, converting the intermediate product dehydroascorbic acid (DHA) to L-ascorbic acid (AsA), thereby lowering the loss and increasing its concentration. Zhang et al. found that glutathione is an antioxidant in cells, and that elevated concentrations of reactive oxygen species (ROS) in tissues cause a decrease in glutathione [30]. Spermine (SP) and spermidine (SPD), which are required for cell division, growth, and apoptosis, are produced when glutathione metabolism-related products are inhibited, which has the benefits of being an antioxidant and antiaging agent. In the β-alanine metabolic pathway, the levels of the differential metabolites spermidine and spermine were elevated. Parthasarathy et al. found that spermine and spermidine, as precursor substances [31], could be converted to β-alanine via 1,3-diaminopropane. In contrast, β-alanine is used in mammals as a strength-enhancing supplement to help promote growth performance and production quality in the organism. In the tyrosine metabolic pathway, the levels of the differential metabolite dopaquinone are elevated. Huang et al. found that dopaquinone, which is involved in tyrosine metabolism [32], can be broken down to form tyrosinase and dopamine. The increase in dopamine content affects the synthesis and secretion of growth hormone (GH) to promote the growth and development of yaks. It can be demonstrated that providing alfalfa to suckling yak calves outweighs providing oat hay in terms of antiaging benefits since it resists cellular oxidation, which can increase productivity and production quality, as well as support healthy body growth.

This study also found significant differences in two metabolic pathways in the group fed oat and alfalfa mixed hay compared to the alfalfa group. In the metabolic pathway of glutathione metabolism, the content of the differential metabolite spermine was significantly decreased. Wu et al. found that glutathione acts as an endogenous antioxidant and fights oxidative stress by scavenging hydroxyl radicals and singlet oxygen molecules [33]. The increase of spermine content can increase the content of glutathione (GSH) in the liver, spleen, and ileum, as well as increase the total antioxidant capacity (TAOC) in them to improve the antioxidant capacity of the body through the nonenzymatic antioxidant system. In the phenylalanine metabolic pathway, the content of the differential metabolite phenylacetylglycine was significantly decreased. Phenylacetylglycine, which is predominantly involved in phenylalanine metabolism and biosynthesis, has been discovered as a possible biomarker for diabetes, according to Wang et al. [34]. The preservation of visceral tissue growth and the reduction in disease damage are both aided by an increase in phenylacetylglycine, which is also actively involved in the control and treatment of metabolic illnesses such as diabetes mellitus. Huang et al. found that phenylalanine is associated with insulin resistance to a lesser extent, and that increased production of phenylacetylglycine precursors by gut microorganisms leads to elevated levels of phenylacetylglycine [35], demonstrating a link between phenylacetylglycine and obesity that may cause changes in glucose and lipid metabolism and the development of diabetes. It can be seen that giving alfalfa hay to lactating yak calves is more advantageous than giving them oat and alfalfa mixed hay because it encourages the mobilization of antioxidants in the viscera, alleviates early growth stress, prevents gastrointestinal uropathy brought on by insulin resistance, and helps to maintain the dynamic equilibrium of glucose and lipid metabolism in the organism. This test result could be a result of the high-quality proteins in alfalfa hay being more effective for yak calves in resisting oxidative stress and illness at the microlevel of metabolomics, where deeper research is needed in the future.

## 5. Conclusions

In comparison to feeding either hay alone, combined feeding oat hay and alfalfa hay was more advantageous to promote morphological and functional development of the pancreas, as well as enhance the digestive metabolism and level of glucose metabolism, thus contributing to an improvement in the growth performance of preweaning yak calves. Therefore, it is suggested oat hay and alfalfa hay combined feeding is an optimized forage supply strategy to healthy and high-quality rearing of preweaning yak calves.

## Figures and Tables

**Table 1:** Nutrient composition of milk replacer powder, concentrates, oat hay and alfalfa hay (dry matter basis) (Unit: %).

Items	Milk Replacer Powder	Concentrates	Oat Hay	Alfalfa Hay
Dry matter (DW)	95.00	87.90	93.00	95.00
Crude protein (CP)	26.24	20.00	4.08	14.19
Ether extract (EE)	27.79	4.70	4.20	3.39
Neutral detergent fiber (NDF)	-	10.90	84.40	64.86
Acid detergent fiber (ADF)	-	4.10	45.18	46.13
Calcium (Ca)	2.50	0.80	1.05	1.55
Phosphorus (P)	1.40	0.45	0.15	0.74

**Table 2:** Effect of different forage source diets on liveweight parameters and the pancreatic index of preweaning yak calves.

Items	OP ^1^	AP ^2^	AOP ^3^	*p*-Value
Initial body weight (kg)	35.8 ± 0.8	36.0 ± 1.6	37.6 ± 0.6	0.053
Average daily gain (g/day)	290.0 ± 3.1	293.3 ± 3.1	310.0 ± 10.0	0.095
Final body weight (kg)	70.6 ± 0.6 ^b^	71.2 ± 1.8 ^b^	74.8 ± 3.1 ^a^	0.017
Dry matter intake (kg/day)	1.00	1.00	1.00	1.000
Pancreas weight (g)	50.34 ± 2.01 ^b^	55.39 ± 2.67 ^ab^	61.44 ± 0.85 ^a^	0.007
Pancreas index (%)	0.07 ± 0.03 ^b^	0.08 ± 0.03 ^b^	0.09 ± 0.03 ^a^	0.022

^a,b^ For the same row of data, different superscript letters denote significant changes (*p* < 0.05), whereas the same superscript letter denotes nonsignificant variations (*p* > 0.05). ^1^ OP represents the group fed oat (n = 5). ^2^ AP represents the group fed alfalfa (n = 5). ^3^ AOP represents the group fed a mixture of oat and alfalfa (n = 5).

**Table 3:** Effect of diets with different forage sources on the share of exocrine and endocrine parts of the total pancreatic area in preweaning yak calves.

Items	OP ^1^	AP ^2^	AOP ^3^	*p*-Value
Share of the total pancreatic endocrine component	0.049 ± 0.001 ^b^	0.047 ± 0.003 ^b^	0.037 ± 0.002 ^a^	<0.001
Share of the total pancreatic exocrine portion	0.87 ± 0.01 ^b^	0.91 ± 0.01 ^a^	0.93 ± 0.01 ^a^	0.009
Area ratio of the exocrine and endocrine pancreas	0.06 ± 0.00 ^b^	0.05 ± 0.00 ^b^	0.04 ± 0.00 ^a^	<0.001

^a,b^ For the same row of data, different superscript letters denote significant changes (*p* < 0.05), whereas the same superscript letter denotes nonsignificant variations (*p* > 0.05). ^1^ OP represents the group fed oat (n = 5). ^2^ AP represents the group fed alfalfa (n = 5). ^3^ AOP represents the group fed a mixture of oat and alfalfa (n = 5).

**Table 4:** Effect of different forage source diets on the activity of pancreatic digestive enzymes in preweaning yak calves (unit: IU/L).

Items	OP ^1^	AP ^2^	AOP ^3^	*p*-Value
Pancreatic amylase	33.8 ± 0.4 ^c^	35.9 ± 0.2 ^b^	36.9 ± 0.2 ^a^	<0.001
Pancreatic lipase	114.7 ± 4.9 ^b^	121.4 ± 3.8 ^ab^	133.2 ± 3.5 ^a^	0.012
Pancreatic protease	163.3 ± 1.1	163.5 ± 2.3	166.7 ± 1.4	0.290
Chymotrypsin	124.6 ± 1.4 ^b^	129.3 ± 6.6 ^ab^	144.4 ± 6.3 ^a^	0.037

^a,b,c^ For the same row of data, different superscript letters denote significant changes (*p* < 0.05), whereas the same superscript letter denotes nonsignificant variations (*p* > 0.05). ^1^ OP represents the group fed oat (n = 5). ^2^ AP represents the group fed alfalfa (n = 5). ^3^ AOP represents the group fed a mixture of oat and alfalfa (n = 5).

**Table 5:** Effect of different forage source diets on pancreatic hormone levels in preweaning yak calves (unit: µg/L).

Items	OP ^1^	AP ^2^	AOP ^3^	*p*-Value
Glucagon	0.30 ± 0.01 ^b^	0.34 ± 0.01 ^a^	0.35 ± 0.01 ^a^	0.002
Insulin	1.08 ± 0.02 ^b^	1.13 ± 0.04 ^ab^	1.20 ± 0.04 ^a^	0.033
Pancreatic polypeptide	2.68 ± 0.11 ^b^	3.02 ± 0.07 ^a^	3.25 ± 0.09 ^a^	0.001
Growth inhibitors	0.29 ± 0.00 ^c^	0.32 ± 0.01 ^b^	0.33 ± 0.00 ^a^	<0.001

^a,b,c^ For the same row of data, different superscript letters denote significant changes (*p* < 0.05), whereas the same superscript letter denotes nonsignificant variations (*p* > 0.05). ^1^ OP represents the group fed oat (n = 5). ^2^ AP represents the group fed alfalfa (n = 5). ^3^ AOP represents the group fed a mixture of oat and alfalfa (n = 5).

**Table 6:** A comparison of the concentrations of metabolites in pancreas tissue from yak calves fed alfalfa hay or oat hay.

No.	Metabolic Pathways	Differential Metabolites	*p*-Value
1	Glutathione metabolism	L-Ascorbate ↑ *; spermidine ↑; spermine ↑	<0.001
2	beta-Alanine metabolism	Spermidine ↑; spermine ↑	0.028
3	Tyrosine metabolism	Dopaquinone ↑	0.037
4	Arginine and proline metabolism	Spermidine ↑; spermine ↑	0.056
5	Ascorbate and aldarate metabolism	L-Ascorbate ↑	0.061
6	HIF-1 signaling pathway	L-Ascorbate ↑	0.061
7	Bile secretion	Spermidine ↑; spermine ↑	0.111
8	Phenylalanine metabolism	Hippuric acid ↓	0.173
9	Tryptophan metabolism	6-Hydroxymelatonin ↓	0.273
10	ABC transporters	Spermidine ↑	0.364
11	Steroid hormone biosynthesis	5β-Androstane-3,17-dione ↓	0.481
12	Vitamin digestion and absorption	L-Ascorbate ↑	0.481
13	Metabolic pathways	Dopaquinone ↑	1.000

* ↑ represents upward adjustment; ↓ represents downward adjustment. OP represents the group fed oat (n = 5). AP represents the group fed alfalfa (n = 5).

**Table 7:** A comparison of the concentrations of metabolites in pancreas tissue from yak calves fed alfalfa and oat hay or oat hay.

No.	Metabolic Pathways	Differential Metabolites	*p*-Value
1	Arginine and proline metabolism	cis-4-Hydroxy-D-proline ↑	0.071
2	Pantothenate and CoA biosynthesis	Pantetheine ↑	0.072
3	Antifolate resistance	dUMP * ↑	0.072
4	Bile secretion	Deoxycholic acid ↓	0.238
5	Pyrimidine metabolism	dUMP ↑	0.268

* dUMP represents uracil deoxyribonucleotide. ↑ represents upward adjustment; ↓ represents downward adjustment. OP represents the group fed oat (n = 5). AOP represents the group fed a mixture of oat and alfalfa (n = 5).

**Table 8:** A comparison of the concentrations of metabolites in pancreas tissue from yak calves fed alfalfa and oat hay or alfalfa hay.

No.	Metabolic Pathways	Differential Metabolites	*p*-Value
1	Glutathione metabolism	Spermine ↓ *	0.040
2	Phenylalanine metabolism	Phenylacetylglycine ↓	0.048
3	beta-Alanine metabolism	Spermine ↓	0.051
4	Arginine and proline metabolism	Spermine ↓	0.071
5	Tyrosine metabolism	Dopaquinone ↓	0.072
6	Bile secretion	Spermine ↓	0.101
7	Metabolic pathways	Spermine ↓	1.000

* ↑ represents upward adjustment; ↓ represents downward adjustment. AP represents the group fed alfalfa (n = 5). AOP represents the group fed a mixture of oat and alfalfa (n = 5).

## Data Availability

All the graphical data from the trial can be found in this article. The raw metabolomics data involved in the text are publicly available and can be found in the MTBLS5503 repository.
